# Differences in Ocular Axial Length Between Genders and Refractive Error Groups: A Cross-Sectional Study at the Yaoundé Central Hospital, Cameroon

**DOI:** 10.7759/cureus.75828

**Published:** 2024-12-16

**Authors:** Joel Gabin Konlack Mekontso, Viola Andin Dohvoma, Steve Robert Ebana Mvogo, Guy Loic Nguefang Tchoukeu, Ulrich Igor Mbessoh Kengne, Fabrice Ndzernyuy Dubila, Fabrice Leo Tamhouo Nwabo, Côme Ebana Mvogo

**Affiliations:** 1 General Medicine, Faculty of Medicine and Biomedical Sciences, University of Yaoundé I, Yaoundé, CMR; 2 Ophthalmology, Faculty of Medicine and Biomedical Sciences, University of Yaoundé I, Yaoundé, CMR; 3 General Medicine, Faculty of Health Sciences, University of Buea, Buea, CMR

**Keywords:** ametropia, cameroon, eye axial length, gender-differences, refractive errors

## Abstract

Background: Refractive errors are a common global health issue. Previous studies in Cameroon have predominantly identified hyperopia and hyperopic astigmatism as the primary refractive errors. This study aimed to determine ocular axial length (OAL) values in Cameroonian adults and to evaluate differences between genders and refractive error groups.

Methods: A cross-sectional study was conducted at the ophthalmology unit of Yaoundé Central Hospital. Participants aged 20-40 years who consented and had no intraocular pathologies or craniofacial malformations were included. OAL was measured using A-scan ultrasonography, and objective refraction was performed following cycloplegia. Age, gender, and refractive error were recorded. Statistical analyses, including Student's t-test, ANOVA, Chi-square, and Pearson correlation coefficient were used with a significance level of p < 0.05.

Results: A total of 200 participants, predominantly female (75.5%), were included. The mean age was 26.36 ± 5.01 years. Men had significantly longer OAL (24.01 ± 0.88 mm) compared to women (23.47 ± 0.84 mm) (adjusted p-value = 0.00). While OAL was slightly larger in myopes and shorter in hypermetropes, these differences were not statistically significant. However, OAL increased significantly with increasing myopia severity (p = 0.000) and decreased with increasing hyperopia severity (p = 0.000). There was a negative, moderate, and significant correlation between OAL and spherical value (r = -0.432 for the right eye, r = -0.429 for the left eye, adjusted p = 0.01).

Conclusion: The OAL values observed in our population were higher than those reported in other studies, and a significant gender difference was noted. These findings suggest that factors other than OAL, such as ocular optics, may significantly influence the hyperopic status of our population.

## Introduction

Refractive errors are common visual conditions that affect hundreds of millions of people of all age groups globally. They are considered a significant public health challenge, as uncorrected refractive errors were the leading cause of visual impairment worldwide in 2010 [1].

The distribution of refractive errors varies across countries [2]. For instance, studies have reported a high prevalence of myopia in East Asian countries [3], whereas hyperopia and hyperopic astigmatism are the most common refractive errors in Cameroonians [4,5].

The refractive power of the eye depends on the interaction between its optical components and its axial length [6]. To our knowledge, no previous studies in Cameroon have measured axial length or explored its relationship with refractive errors. This study aims to establish normative values for axial length in a population of Cameroonian adults, identify differences between genders and refractive error groups, and compare these values with those reported in other populations.

## Materials and methods

Study design and setting

We conducted a four-month cross-sectional study in the Ophthalmology Unit of Yaoundé Central Hospital from January to May 2017. This public hospital is well-attended and serves patients of diverse social backgrounds from all regions of Cameroon, providing a representative sample of the country's population.

Study population

We used consecutive sampling and included all consenting patients aged 20-40 years who attended consultations during the study period. We excluded monocular patients and those with a history of orbital trauma or disease, endocrine disorders, congenital glaucoma, or craniofacial malformations.

Data collection

Ophthalmic examinations included uncorrected distance visual acuity testing, slit-lamp examination, automatic refraction under cycloplegia, and fundoscopy with a 78D lens. Cycloplegia was achieved by alternately instilling one drop of cyclopentolate 0.5% and one drop of tropicamide 0.5% at five-minute intervals, for a total of three drops per agent. Refraction was measured 20-30 minutes after the final drop.

Ocular axial length was measured by a single operator using the A-scan contact technique with the SONOMED E-Z Scan AB5500 (Spectrum Ophthalmics Inc., Woodinville, WA, USA). Corneal anesthesia was achieved by instilling two drops of 0.4% oxybuprocaine hydrochloride in the inferior conjunctival sac one minute before measurement. The mean of five consecutive measurements (with a standard deviation <0.1 mm) was recorded for each eye.

Definitions

Eyes with spherical values (SV) ≤ -0.25D or ≥ +0.25D were classified as myopic or hyperopic, respectively. Astigmatism was defined as a cylindrical value ≥ 0.50D. Refractive errors were then categorized as hyperopia, myopia, hyperopic astigmatism, myopic astigmatism, or mixed astigmatism [4]. Further classifications included mild, moderate, and high myopia as a SV of −0.5 to −3.0D, −3.1 to −6.0D, and less than −6.0D, respectively. To classify hyperopia, we used SV ranges of 0.5 to 3.0D, 3.1 to 6.0D, and more than 6.0D for mild, moderate, and high hyperopia, respectively.

Statistical analysis

We collected data on age, sex, objective refraction, and OAL. Data analysis was performed using SPSS version 23.0 (IBM Corporation, Armonk, NY, USA). We applied Student's t-test, ANOVA, Chi-square, and Pearson correlation coefficient as appropriate, with statistical significance set at p < 0.05.

Ethical considerations

The study was reviewed and approved by the Institutional Review Board of the Faculty of Medicine and Biomedical Sciences, University of Yaoundé I (approval ID: 330/UY1/FMSB/VDRC/CSD). Written informed consent was obtained from all participants, and authorization was secured from the manager of the participating health facility.

## Results

**Figure 1:**
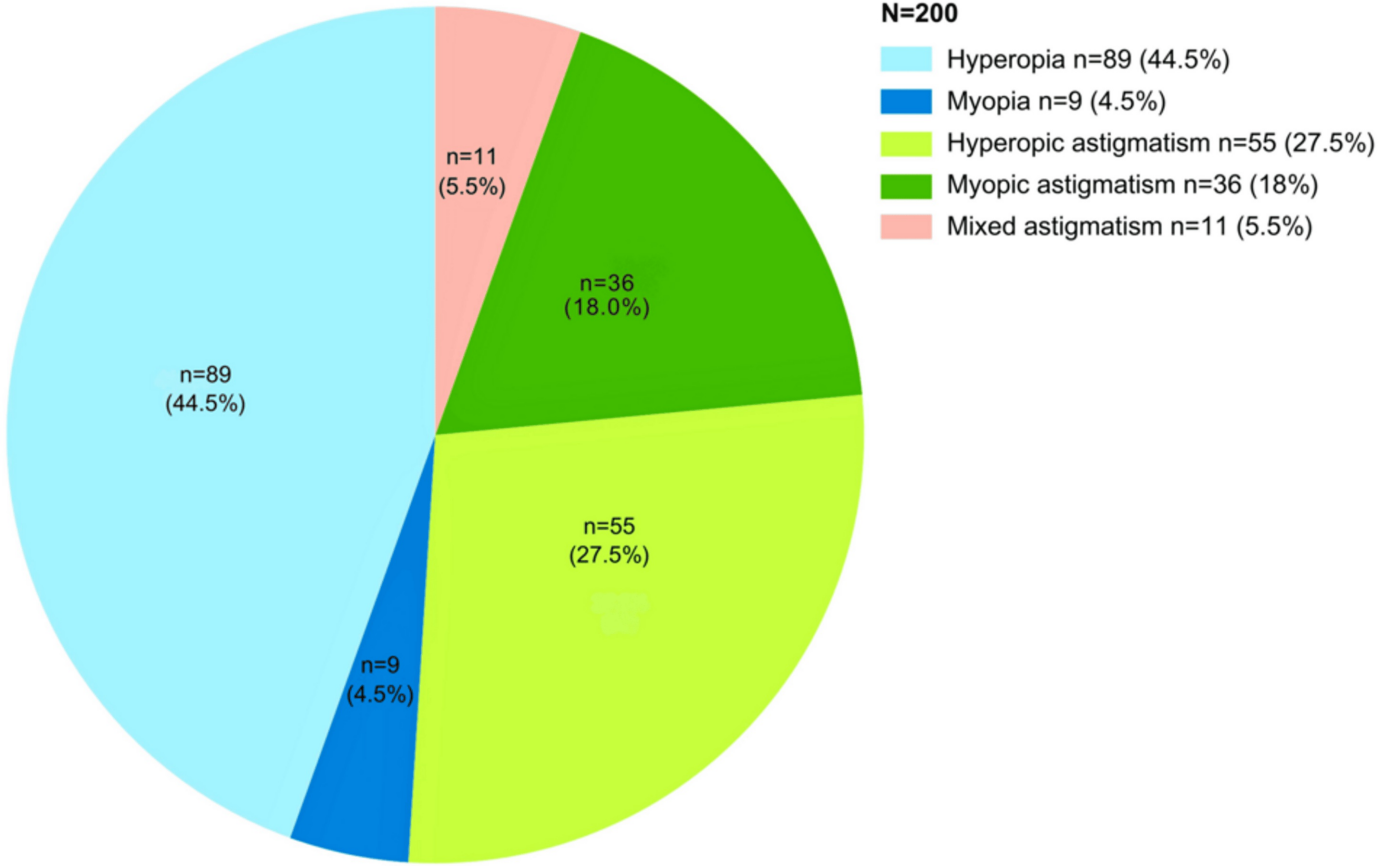
Distribution of the study population with respect to refractive errors

A total of 200 patients aged 20 to 40 years were included in the study, with 75.5% (n = 151) being female. The mean age was 26.36 ± 5.01 years. Hyperopia was the most common refractive error (44.5%), followed by hyperopic astigmatism (27.5%). Figure 1 illustrates the distribution of refractive errors among the study population. No significant differences were observed in the distribution of refractive errors based on laterality, age, or sex (Chi-square test, p = 0.43, p = 0.06, and p = 0.19, respectively).

OAL values ranged from 20.52 mm to 28.49 mm in both eyes, with a mean of 23.60 ± 0.90 mm in the right eye and 23.60 ± 0.86 mm in the left eye (t-test, p = 0.96). The mean OAL was significantly greater in men (24.01 ± 0.88 mm) compared to women (23.47 ± 0.84 mm) (t-test, adjusted p = 0.00) and did not vary significantly between different age groups (ANOVA, p = 0.323 for the right eye and p = 0.372 for the left eye).

OAL decreased progressively from myopic astigmatism to hyperopic astigmatism (Table 1). However, there were no statistically significant differences in OAL across the refractive error groups (ANOVA, adjusted p = 0.66).

**Table 1:** Mean and standard deviation of ocular axial length values with respect to the type of refractive error

	Minimum (mm)	Maximum (mm)	Mean (mm)	Standard deviation (mm)
Myopic astigmatism	22.54	28.49	24.44	1.12
Myopia	22.69	24.83	23.59	0.53
Mixed astigmatism	22.50	24.40	23.58	0.55
Hyperopia	21.93	24.90	23.45	0.69
Hyperopic astigmatism	20.52	24.86	23.30	0.74

A significant trend in OAL was observed within the severity of refractive errors: OAL increased with the severity of myopia (ANOVA, p = 0.000) and decreased with the severity of hyperopia (ANOVA, p = 0.000) (Table 2).

**Table 2:** Distribution of ocular axial length according to the severity of myopia and hyperopia

	Minimum (mm)	Maximum (mm)	Mean (mm)	SD (mm)
Mild myopia	22.54	25.33	23.93	0.64
Moderate myopia	24.64	25.88	25.38	0.42
High myopia	26.49	28.49	27.27	0.76
Mild hyperopia	21.93	24.90	23.42	0.66
Moderate hyperopia	22.30	23.72	23.22	0.79
High hyperopia	20.65	21.11	20.76	0.31

**Figure 2:**
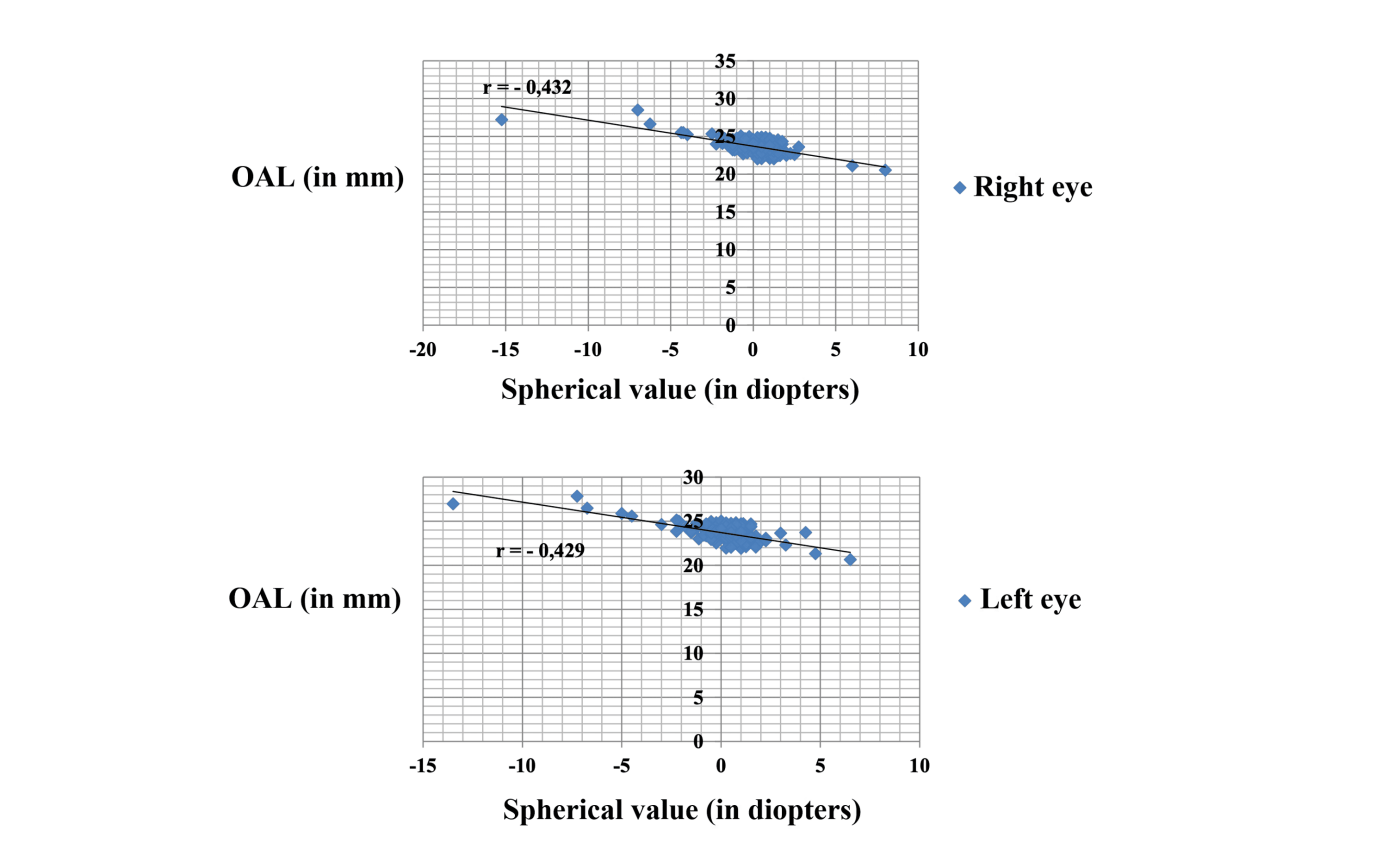
Correlation between the ocular axial length and spherical value OAL = ocular axial length

Overall, there was a negative, moderate and significant correlation between OAL and SV (Figure 2) (Pearson correlation coefficient r = -0.432 for the right eye, r = -0,429 for the left eye, adjusted p=0.01).

## Discussion

This study aimed to determine the OAL values in Cameroonian adults and examine their distribution by gender and across various refractive error groups. Given the hospital-based nature of the study, we adopted lower thresholds to define refractive errors compared to other authors. We used a combination of cyclopentolate and tropicamide to achieve optimal cycloplegia because Blacks tend to have darker iris pigmentation and often exhibit a stronger accommodative response. This approach was necessary to ensure accurate refractive measurements [4].

Eye growth patterns with age are well-documented in the literature. Studies have shown that nearly half of postnatal eye growth occurs within the first 12-16 months of life, with elongation decelerating and generally ceasing by the age of 13 years [7]. Other findings suggest that ocular growth may continue until 18 years of age [7,8]. For this reason, our lower age limit was set at 20 years. To minimize the impact of age-related refractive changes, participants were limited to those under 40 years old. After the fourth decade, oxidative damage due to a decline in glutathione transport accelerates lens degeneration. This process leads to decreased transparency and increased light scattering, contributing to conditions like presbyopia and cataracts. [9]. Therefore, our study population, aged 20-40 years, was relatively homogenous regarding growth and aging processes.

The most prevalent refractive error in our study was hyperopia, followed by hyperopic astigmatism, aligning with previous studies in Cameroonian non-albino populations by Mvogo et al. [4] and Dohvoma et al. [5]. In contrast to Cameroonian albino populations, where myopic astigmatism predominates [10], our findings suggest a potential role of melanin in influencing refractive error types. Racial differences in the prevalence of refractive errors have been widely reported [11-13]. Neither sex nor age significantly influenced the distribution of refractive errors in our study, a finding consistent with some studies [4,5,14] but contrasting with others that associate female sex with a higher risk of myopia [15]. These differences are potentially explained by variations in study methods, definitions, and demographics. The lower male participation in our study may be due to societal factors, but the hospital-based design, reflecting real-world burden of refractive errors, and lack of gender association support generalizability.

The mean OAL in men was significantly longer than in women, potentially reflecting height differences between genders [16]. This observation aligns with findings from several authors [17,18], although others have reported no significant differences [19]. Such discrepancies might be explained by racial, genetic, or methodological variations across populations. Ocular axial length remained stable across age groups in this study, corroborating findings by Bhardwaj et al., who reported a decline in OAL only after the fourth decade [8]. Understanding gender differences in OAL is crucial for optimizing surgical outcomes, particularly in procedures like cataract surgery. Precise intraocular lens power calculations rely on accurate biometric measurements, and accounting for gender-specific differences in OAL can significantly improve postoperative visual outcomes.

Our results confirm that myopes tend to have longer OALs, while hyperopes have shorter OALs, consistent with previous studies [8]. The differences in OAL across refractive error groups were not statistically significant, likely due to the small sample size. However, OAL significantly increased with greater myopia severity and decreased with increasing hyperopia severity. This supports findings by Touzeau et al., who identified OAL as a critical parameter strongly correlated with SVs, playing a central role in the genesis of spherical ametropias, while corneal toricity was primarily responsible for astigmatism [6].

The mean OAL in our study appears significantly higher than those reported by other authors [17,18,20-28]. This discrepancy may be attributed to the fact that many of these studies were conducted on patients undergoing cataract surgery, most of whom were over 60 years old. Notably, OAL decreases significantly after the age of 40, with a reduction of approximately 0.40 mm compared to younger individuals [8]. Racial, ethnic, and genetic differences, likely linked to variations in facial structure and ocular anatomy, may also contribute to these discrepancies [20,28]. Although the predominantly hyperopic status of the Cameroonian population might suggest shorter OALs, our findings suggest that OAL alone does not fully account for the distribution of refractive errors in this group. Further exploration of ocular optics and other biometric parameters is needed.

This study's brief duration and small sample size may have limited the statistical power to detect significant differences in OAL across refractive error groups. Additionally, the lack of data on other critical ocular biometric parameters, such as keratometry, corneal, and lens thickness, restricted a more detailed understanding of how these elements influence the population's hyperopic profile. Future studies with larger cohorts and comprehensive biometric evaluations could offer deeper insights into the relationships between ocular biometry and refractive errors in this population.

## Conclusions

The OAL in our population is significantly greater in men than in women and slightly higher than values reported in other populations. Myopes tend to have slightly longer eyes, while hypermetropes have shorter eyes; however, the differences across various refractive error groups are not statistically significant. OAL varies significantly with the severity of static ametropias. These findings suggest that OAL alone does not fully explain the distribution of refractive errors in our population. Future studies with a larger sample size and the inclusion of other ocular biometric parameters could provide a better understanding of how ocular biometry in Cameroonians correlates with the predominantly hyperopic status of this population.
